# Weighted Zernike defocus coefficient of treatment zone is a meaningful indicator for myopia control efficacy of Ortho-K lenses

**DOI:** 10.1186/s40662-022-00296-0

**Published:** 2022-07-01

**Authors:** Yuzhuo Fan, Yan Li, Kai Wang, Jia Qu, Mingwei Zhao

**Affiliations:** 1 Institute of Medical Technology, Peking University Health Science Center, Beijing, 100191 China; 2 Department of Ophthalmology & Clinical Center of Optometry, Peking University People’s Hospital, Beijing, 100044 China; 3 College of Optometry, Peking University Health Science Center, Beijing, China; 4 Eye Disease and Optometry Institute, Peking University People’s Hospital, Beijing, China; 5 Beijing Key Laboratory of Diagnosis and Therapy of Retinal and Choroid Diseases, Beijing, China; 6 School of Ophthalmology and Optometry and Eye Hospital, Wenzhou Medical University, Wenzhou, Zhejiang China

**Keywords:** Myopia, Orthokeratology, Axial length, Defocus, Zernike

## Abstract

**Background:**

The goal of this study was to reproduce a three-dimensional representation of corneal defocus characteristics after orthokeratology (Ortho-K) treatment via an indicator defined as the weighted Zernike defocus coefficient of the treatment zone (*C*_*weighted defocus*_). This could be used to predict the effectiveness of Ortho-K treatment quantitatively in a timely manner after the one-month visit.

**Methods:**

Seventy myopic children with axial length (AL) elongation after Ortho-K treatment (group A) and 63 myopic children with AL shortening after Ortho-K treatment (group B) were included in this one-year retrospective study. The proposed indicator was calculated by a customized MATLAB program. Multivariate binomial logistic regression and multivariate linear regression analyses were used to explore the association between AL change and the *C*_*weighted defocus*_, age, sex, and other ocular biometric parameters.

**Results:**

The 12-month AL change, age, pupil diameter, and vertical decentration of the Ortho-K lens were significantly different between the two groups. Multivariate logistic regression analysis showed that a larger *C*_*weighted defocus*_ (≥ 0.35 D/mm^2^) (OR: 0.224; 95% CI: 0.078–0.646; *P* = 0.006) was correlated with the emergence of AL shortening after orthokeratology treatment. A multivariate linear regression model showed that a greater *C*_*weighted defocus*_ was associated with slower 12-month AL elongation (β =  − 0.51, *P* = 0.001).

**Conclusions:**

The *C*_*weighted defocus*_ is an effective predictive indicator of myopia control, and a larger *C*_*weighted defocus*_ may lead to slower elongation of AL. This meaningful indicator may help in the evaluation and adjustment of Ortho-K lens parameters in a timely manner and minimize the cost of clinical trial and error.

## Background

In recent years, the prevalence of myopia has increased worldwide, and this condition tends to be identified at a young age [1]. Myopia progression is accompanied by an increase in the axial length (AL), and once it develops into high myopia, the risk of vision-threatening complications from longer AL is greatly increased [2, 3]. Therefore, there is an urgent need to control the progression of myopia in children and adolescents.

Ortho-K is currently the most clinically effective optical means for myopia control, and its effectiveness (33–56% myopia progression reduction over a two-year treatment period) has been confirmed by many studies [4–7]. At present, the peripheral defocus hypothesis is the mechanism by which Ortho-K is theorized to control myopic progression. Alterations in corneal refractive power modulate visual input signals, induce relative myopic defocus in the peripheral retina and slow AL elongation [8–11].

In recent years, many studies have attempted to quantitatively assess the effectiveness of Ortho-K for AL control based on the defocus characteristics produced during the treatment. However, the methods of existing studies remain incomprehensive as they consider only certain axis directions [12, 13], specific regions [14, 15] or profiles [16] and do not fully reflect the morphology of the cornea in its entirety and the overall defocus characteristics in a three-dimensional space. In addition, it has been demonstrated that Ortho-K for myopia control significantly increases the Zernike coefficients and ocular higher-order aberrations in children [17, 18]. Some studies have reported that Ortho-K designs with a smaller treatment area can enhance exposure to peripheral defocus and higher-order aberrations, with less AL growth. Meanwhile, increasing the Ortho-K compression factor by 1.00 D significantly altered some higher-order aberrations [19].

Therefore, to obtain a more comprehensive consideration, we reconstructed the three-dimensional Zernike defocus values within the treatment zone after Ortho-K lens treatment and defined it as the weighted Zernike defocus coefficient of the treatment zone **(***C*_*weighted defocus*_**)** by Zernike fitting, which realistically reproduces the defocus characteristics of the entire treatment zone by considering the size of the treatment zone; in this study, the pupil size and lens decentration were also considered. In other words, this indicator combines several parameters previously reported to be effective for AL control, such as treatment area [20, 21], Jessen factor [19], and defocus volume of Ortho-K [15], to create a more comprehensive and effective predictive index. We expect that this index can truly and quantitatively evaluate the defocus characteristics in the real treatment zone, and we further analyze whether this index plays an important role in slowing the elongation of AL. We also expect that the control efficacy of Ortho-K treatment can be quantitatively evaluated and that this index can be used as an adjustable indicator to guide Ortho-K lens fitting in the future.

## Methods

### Study participants

All patients were retrospectively enrolled from the clinical case files of myopic children who were fitted with Ortho-K lenses [corneal refractive therapy (CRT) and vision shaping treatment (VST) lens design] from January 2018 to December 2019 in the Optometry Centre of Peking University People's Hospital. A total of 133 patients who met the recruitment criteria were recruited for the study. To eliminate correlative effects between the two eyes, only data from the right eyes were collected in this study.

The inclusion criteria were as follows: (1) no history of using atropine eye drops, (2) a best-corrected visual acuity (BCVA) value of no less than 20/20 for either eye, (3) a cycloplegic spherical power of no less than − 0.75 diopters (D) and astigmatism no greater than 3.00 D, and (4) no prescription modification during Ortho-K lens wear follow-up. Patients with any other ocular diseases or poor topographic measurement quality were excluded from the study. The final parameters of the ordered lenses were confirmed by both fluorescein staining at lens delivery and an ideal “bull’s eye” corneal topography pattern after lens treatment. No severe corneal complications were observed. The purpose and details of the study were explained to all the subjects and their parents or guardians, and signed informed consent was obtained. This study followed the tenets of the Declaration of Helsinki and was approved by the Medical Ethics Committee of Peking University People’s Hospital.

### Ocular parameters and measurement method

The data collected in this study included age at the initial visit, sex, baseline spherical and cylindrical refraction, initial mean keratometry (K) readings, corneal eccentricity (E value), horizontal visible iris diameter (HVID), anterior chamber depth (ACD), lens design (VST or CRT), treatment zone decentration, pupil diameter and 12-month AL change (AL at the 12-month follow-up minus baseline AL). Corneal parameters were obtained using a Sirius corneal topography system (CSO, Italy), and the pupil diameter was measured using the Sirius device with the examination room lights off (room illuminance 2 lx). The.csv files of the corneal sagittal height data (at baseline and one-month after Ortho-K treatment) were exported from the Sirius system. An IOLMaster (Carl Zeiss, Ltd., Germany) was used to measure the AL of all subjects, and the results for data analysis were obtained by averaging six repeated measurements in which intrasession differences were no greater than 0.02 mm. Cycloplegic refraction was measured 35 min after three instillations of 1% cyclopentolate administered at five-minute intervals, and spherical and astigmatic refraction readings were obtained using an autorefractor. The spherical equivalent (SE) was calculated as the spherical power plus 1/2 cylindrical power.

### Calculation of the weighted Zernike defocus coefficient of the treatment zone

**Fig. 1:**
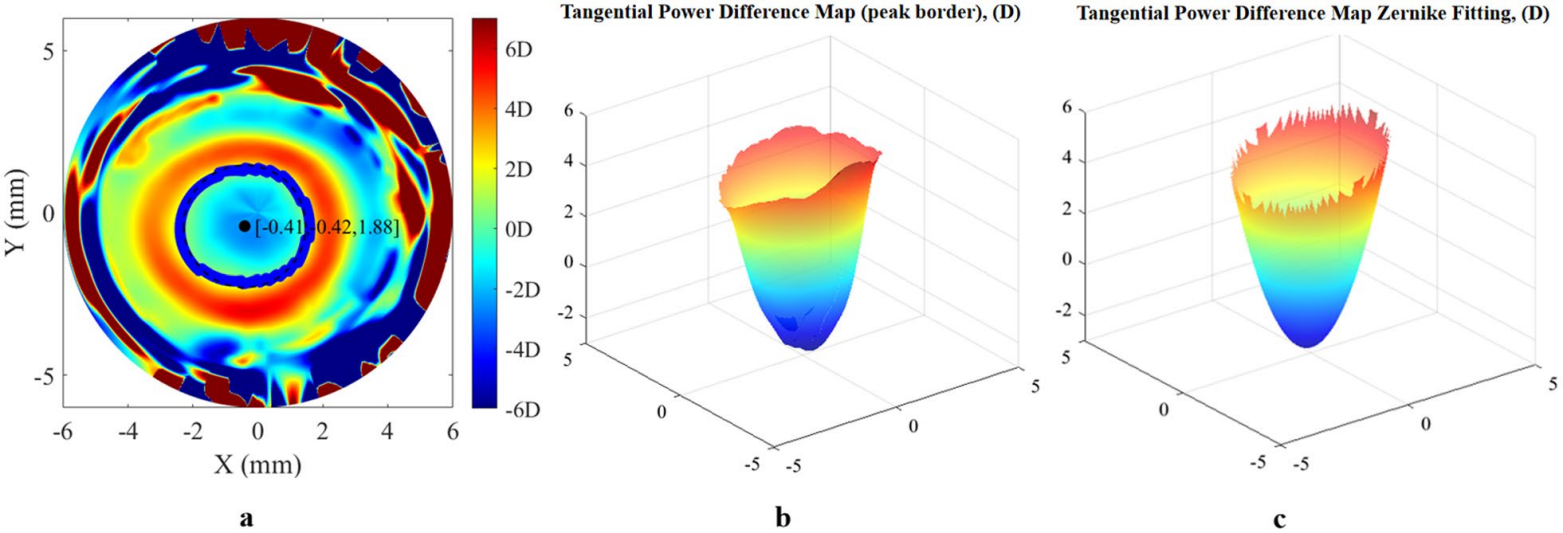
Reconstruction of tangential power difference map after Ortho-K treatment. **a** Two-dimensional original tangential power difference map; **b** Real tangential power difference map in three-dimensional space; **c** Reproduction of tangential power difference map by Zernike fitting

Clinically, the tangential power difference map after Ortho-K treatment was a bowl-like shape in three-dimensional space, which can be generated by minus tangential power map after Ortho-K treatment from the initial visit (Fig. 1). The raw data of these two maps were exported from the Sirius corneal topography system as two 31 × 256 matrices. On this difference map, the data within the maximal positive defocus border was extracted, and the matrix of tangential power difference (D) was calculated using:

$${T}_{diff}(\rho ,\theta ) = {T}_{post}(\rho ,\theta )-{T}_{pre}(\rho ,\theta )$$, where $$\rho \epsilon [\mathrm{0,1}], \theta \epsilon [\mathrm{0,2}\pi ]$$

where *ρ* is the power of the radial defocus ring coordinate, and the border is defined as the maximal positive defocus point on the tangential difference map; *θ* is the azimuthal angle; *T* is the matrix of tangential corneal power; and ${T}_{diff}(\rho ,\theta )$, ${T}_{pre}(\rho ,\theta )$ and ${T}_{post}(\rho ,\theta )$ are the matrices of the tangential power difference map and the tangential power maps before and after Ortho-K treatment, respectively. A Zernike fitting was then performed to calculate the Zernike defocus coefficient (${C}_{2}^{0}$, D), which can be expressed as [22]:

$${C}_{2}^{0} = \frac{1}{\pi }{\int }_{0}^{1}{\int }_{0}^{2\pi }{T}_{diff}(\rho ,\theta )\sqrt{3}(2{\rho }^{2}-1)\rho d\rho d\theta$$

Then, the weighted Zernike defocus coefficient of the treatment zone (*C*_*weighted defocus*_, D/mm^2^) can be defined as:

$${C}_{weighted defocus} = \frac{{C}_{2}^{0}}{\pi \cdot {r}^{2}}$$

To calculate the treatment zone area (π·*r*^2^), 256 points on the treatment zone border, which were defined as the points of transition from negative to positive values, were automatically extracted from this tangential difference map by a customized MATLAB program. The least squares method was then used to estimate the best fitting “ring” for these points. The treatment zone area can be calculated as π·*r*^2^ (*r*: radius of the best fitted ring), and the decentration of the treatment zone can also be deduced from this ring.

### Statistical analysis

SPSS software (version 19.0, SPSS Inc. Chicago, IL, USA) was used for statistical analysis. The Shapiro-Wilk test was used to examine the normality of the data. Means and standard deviations or counts and percentages are reported as appropriate. The clinical characteristics were compared between groups A and B using independent t-test, a Mann-Whitney *U* test, a χ^2^ test, or Fisher's exact test as appropriate.

Univariate and multivariate binary logistic regression were used to analyze the correlations of AL elongation (> 0 defined as yes; ≤ 0 defined as no) with age, sex, *C*_*weighted defocus*_ (large *C*_*weighted defocus*_ was defined as an index ≥ 0.35 D/mm^2^ based on the median of the calculated data), treatment zone decentration, lens design and pupil diameter. The confounding factors were defined as clinical variables with *P* values less than 0.05 in the comparison analysis. A two-tailed *P* value less than 0.05 was considered as statistically significant.

The Pearson correlation coefficient was used to evaluate the simple correlations between 12-month AL elongation and the *C*_*weighted defocus*_ of the treatment zone. The association was further examined using linear regression analyses. Variables, including the baseline age, sex, SE, horizontal decentration, vertical decentration, pupil diameter, mean K, HVID, E value, and *C*_*weighted defocus*_, were first examined using univariate linear regression analysis. Variables that showed statistically significant associations (*P* < 0.05) with the 12-month AL change in univariate analyses were included in the multivariate regression model. These variables included baseline pupil diameter, age, SE, horizontal decentration and *C*_*weighted defocus*_ of the treatment zone.

## Results

### Description of clinical data

A total of 133 patients (133 right eyes) were involved in the study, including those with an AL change > 0 mm (n = 70) and those with an AL change ≤ 0 mm (n = 63). All patients (mean age: 11.08 ± 2.01 years) underwent successful Ortho-K treatment without atropine eye drops. Sixty-three (47.37%) patients were male. The basic demographics of the subjects is shown in Table 1.

**Table 1:** Clinical characteristics of the study population

Parameters	Mean ± SD, n (%)			*P* value
	All patients (n = 133)	Axial length growth Group A (n = 70)	Axial length shortening Group B (n = 63)	
Age (years)	11.08 ± 2.01	10.28 ± 1.59	11.97 ± 2.06	< 0.001
Sex, male (%)	63 (47.37)	32 (45.71)	31 (49.21)	0.69
12-month axial length change (mm)	0.04 ± 0.22	0.20 ± 0.15	− 0.15 ± 0.09	< 0.001
*C*_*weighted defocus*_ (D/mm^2^)	0.39 ± 0.17	0.31 ± 0.11	0.47 ± 0.18	< 0.001
Horizontal decentration (mm)	− 0.09 ± 0.54	− 0.18 ± 0.50	0.00 ± 0.57	0.06
Vertical decentration (mm)	− 0.19 ± 0.40	− 0.14 ± 0.37	− 0.26 ± 0.42	0.03
Design, VST (%)	56 (42.11)	29 (41.43)	27 (42.86)	0.87
SE (D)	− 3.51 ± 1.64	− 3.10 ± 1.34	− 3.99 ± 2.04	0.002
Pupil diameter (mm)	3.92 ± 0.58	3.67 ± 0.51	4.19 ± 0.53	< 0.001
K mean (D)	43.33 ± 1.36	43.30 ± 1.26	43.36 ± 1.47	0.79
HVID (mm)	11.98 ± 0.43	12.03 ± 0.45	11.92 ± 0.41	0.13
E value	0.51 ± 0.10	0.51 ± 0.10	0.51 ± 0.11	0.80
ACD (mm)	3.36 ± 0.21	3.36 ± 0.20	3.37 ± 0.23	0.76

*VST* = vision shaping treatment; *SE* = spherical equivalent; *K* = keratometry; *HVID* = horizontal visible iris diameter; *ACD* = anterior chamber depth

### Assessment of differences between the two groups

After analyzing and comparing the demographic data and ocular parameters in this study, the results indicated statistically significant differences in age, pupil diameter, and vertical decentration between the two groups. Among these variables, as shown in Table 1, the group with shortened AL (group B) was older and had more vertical decentration, a greater myopia SE and a greater *C*_*weighted defocus*_ of the treatment zone. There were no statistically significant differences in sex, horizontal decentration, K mean, HVID, E value or ACD in this study (*P* > 0.05) between groups A and B.

### Logistic regression analysis

Univariate logistic regression analysis showed that a larger *C*_*weighted defocus*_ of the treatment zone (≥ 0.35 D/mm^2^) (OR: 0.199; *P* < 0.001), older age (OR: 0.596; *P* < 0.001), larger pupil diameter (OR: 0.135; *P* < 0.001) and more myopic SE (OR: 1.449; *P* = 0.002) were correlated with the emergence of AL shortening after Ortho-K treatment (Table 2).

**Table 2:** Logistic regression analysis of the associations of the 12-month axial length change with basic and ocular characteristics

Parameters	Univariate logistic regression			Multivariate logistic regression*		
	OR	95% CI	*P*-value	OR	95% CI	*P* value
*C*_*weighted defocus*_ (D/mm^2^)	0.199	0.016, 0.098	< 0.001	0.224	0.078, 0.646	0.006
Age (years)	0.596	0.473, 0.750	< 0.001	0.585	0.432, 0.793	0.001
Pupil diameter (mm)	0.135	0.059, 0.309	< 0.001	0.116	0.041, 0.323	< 0.001
SE (D)	1.449	1.115, 1.829	0.002	1.157	0.820, 1.631	0.406
Sex, male	0.869	0.440, 1.719	0.687	1.262	0.467, 3.410	0.647
Lens design, VST	0.943	0.473, 1.879	0.868	1.098	0.420, 2.871	0.849
Vertical decentration (mm)	2.159	0.886, 5.265	0.091	5.127	1.404, 18.730	0.013

*SE* = spherical equivalent; *VST* = vision shaping treatment; *OR* = odds ratio; *CI* = confidence interval

*Adjusted for *C*_*weighted defocus*_, sex, age, pupil diameter, SE, lens design, and vertical decentration

Multifactorial logistic regression analysis was used to assess the effects of age, sex, treatment zone decentration, *C*_*weighted defocus*_, lens design, SE, and pupil diameter on the elongation of AL in the study subjects. The model was statistically significant and was able to correctly classify 80.5% of the study subjects (χ^2^ = 71.26,* P* < 0.001). The results showed that after adjusting for age, sex, pupil diameter lens design and treatment zone decentration, a larger *C*_*weighted defocus*_ (≥ 0.35 D/mm^2^) remained correlated with the emergence of AL shortening after Ortho-K treatment (OR: 0.224; *P* = 0.006; Table 2). Baseline SE was associated with AL elongation in the univariate analysis. The association became statistically nonsignificant in the multivariate regression model.

### Linear regression analysis

The 12-month AL elongation showed a significant correlation with the *C*_*weighted defocus*_ of the treatment zone (R =  − 0.445, *P* < 0.001). As shown in Table 3, age, pupil diameter, horizontal decentration, SE and *C*_*weighted defocus*_ were significantly associated with 12-month AL elongation in univariate linear regression. After further analysis, age, pupil diameter and *C*_*weighted defocus*_ of the treatment zone were significantly associated with 12-month AL elongation in multivariate linear regression analyses ('enter' method), and the regression models were statistically significant (R = 0.636, *P* < 0.001; Table 3). A greater *C*_*weighted defocus*_ of the treatment zone was associated with slower AL elongation at the 12-month visit (β =  − 0.51, *P* = 0.001).

**Table 3:** Linear regression analysis of the associations of the 12-month axial length change with basic and ocular characteristics

Parameters	Univariate regression			Multivariate regression**		
	R	Beta (95% CI)	*P* value	Beta (95% CI)	*P* value	Standardized beta
Age (years)	0.437	− 0.047 (− 0.064 to − 0.031)	< 0.001	− 0.035 (− 0.051 to − 0.020)	0.001	− 0.325
Sex, male	0.085	0.037 (− 0.038 to 0.112)	0.328			
*C*_*weighted defocus*_ (D/mm^2^)	0.445	− 0.581 (− 0.784 to 0.379)	< 0.001	− 0.510 (− 0.732 to − 0.288)	0.001	− 0.391
Horizontal decentration (mm)	0.170	− 0.069 (− 0.138 to 0.001)	0.050	− 0.040 (− 0.096 to 0.015)	0.156	− 0.99
Vertical decentration (mm)	0.007	0.004 (− 0.091 to 0.098)	0.941			
SE (D)	0.201	0.027 (0.004 to 0.049)	0.020	− 0.018 (− 0.041 to 0.004)	0.107	− 0.139
Pupil diameter (mm)	0.400	− 0.151 (− 0.210 to − 0.091)	< 0.001	− 0.094 (− 0.148 to − 0.041)	0.001	− 0.250
Mean K (D)	0.009	0.001 (− 0.026 to 0.029)	0.920			
HVID (mm)	0.058	0.030 (− 0.058to 0.117)	0.853			
E value	0.003	− 0.006 (− 0.378 to 0.365)	0.973			
ACD (mm)	0.112	− 0.114 (− 0.289 to 0.061)	0.211			

*SE* = spherical equivalent; *K* = keratometry; *HVID* = horizontal visible iris diameter; *ACD* = anterior chamber depth; *CI* = confidence interval

**Inclusion of variables: age, *C*_*weighted defocus*_, horizontal decentration, pupil diameter, SE (R = 0.636, R^2^ = 0.405, Adjusted R^2^ = 0.381, *P* < 0.001)

## Discussion

Ortho-K has been demonstrated to be an effective means of myopia control in many studies [4–7]. However, a small percentage of patients remain insensitive to defocus and do not respond to Ortho-K with respect to slowing of axial elongation [14, 23], and thus such patients could be a challenge for clinical practitioners. Currently, a follow-up period of at least six months to a year is generally required to assess the effect of Ortho-K in myopia control. If possible, earlier assessment and prediction as well as timelier lens parameter adjustments will allow better myopia control effects of Ortho-K treatment.

Most previous studies, although limited, have demonstrated that the positive defocusing characteristics of Ortho-K lenses are correlated with elongation of the AL [11, 12, 15, 16]. Therefore, we further performed corneal three-dimensional reconstruction to develop an overall Zernike model of the treatment zone after Ortho-K treatment and extracted the defocus terms from this model to realistically reproduce the defocus characteristics of the treatment zone. Here, the influence of various factors, such as treatment zone decentration, area, and pupil size were considered. In addition, during the modelling, both the three-dimensional spatial structure and the most realistic location and size of the treatment zone after Ortho-K treatment were considered.

In the construction of both the binary logistic regression model and the multiple linear regression model, three variables – age, *C*_*weighted defocus*_, and pupil diameter – consistently showed significant correlations with the amount of AL growth over one year. The pupil diameter, measured in the same light environment in all patients, was collected in this study, and its mean value was larger in the axial shortening group (group B). The reason for this could be that a larger pupil might be accompanied by a greater defocus volume in the pupil, after which the retina would receive a more peripheral myopic defocus signal induced by Ortho-K as a protective factor for myopia control [8, 21, 24, 25]. In the current study, a greater baseline myopic SE was related to slower axial growth in univariate regression analysis. However, the association became statistically insignificant after multivariate regression.

This study did not restrict the designs or brands of the Ortho-K lenses used, demonstrating that *C*_*weighted defocus*_ of the treatment zone is applicable to all brands and designs. Corneal responses can vary based on the Ortho-K lens design as reported in the literature [26, 27]. Considering the small group sizes in our study, we cannot conclude that the different Ortho-K lens design or brand have identical effects on the cornea after Ortho-K treatment. We can only go so far to say that lens design was not a major influencing factor in this study and would not have affected our findings.

However, some myopia control effects of Ortho-K are unpredictable and cannot be evaluated by various prediction models. These effects may be more related to duration of near work, genetics, intelligence quotient and other factors [28–31]. More research could be performed in the future to explore and explain these effects.

The *C*_*weighted defocus*_ is useful for quantitatively assessing the control effect of Ortho-K lenses. This index can clarify the correlation between the defocus characteristics of the treatment zone after Ortho-K treatment and the myopia control effect in adolescents, and thus predicts the rate of myopia progression early on and allows the application of further interventions. We found that the greater the baseline SE is, the greater is the *C*_*weighted defocus*_. Furthermore, during the design of the Ortho-K lens parameters, we can increase the *C*_*weighted defocus*_ by reducing the treatment area as much as possible. Increasing the Jessen factor within the allowable range can also increase *C*_*weighted defocus*_. It is hoped that in the future, the index will not only be used to quantitatively assess the control effect of Ortho-K but also act as an adjustable index to guide the fitting process for Ortho-K lenses. The index, together with the model, can even analyze and evaluate patients who have already undergone multiple myopia control measures, such as those who have been treated with a combination of Ortho-K lenses and atropine eye drops. If the *C*_*weighted defocus*_ has been found to be a more desirable result (≥ 0.35 D/mm^2^), it serves as evidence that the prescription of the Ortho-K lens is suitable. In this case, it may be that other myopia control procedures, such as the existing concentration or duration of use of low concentration atropine eye drops, need to be adjusted.

One limitation of this study is that the sample size is small; we will consider expanding the sample size to improve the effectiveness of the prediction efficacy of the model in the future. This study was also performed in retrospective, so further consideration will be given to the possibility of conducting a prospective study to evaluate the validity of this *C*_*weighted defocus*_ index in predicting the effectiveness of Ortho-K lenses for myopia control.

## Conclusions

In conclusion, the weighted Zernike defocus coefficient of the treatment zone effectively predicted the effectiveness of Ortho-K for AL control after one month visit. Ortho-K lenses with a greater *C*_*weighted defocus*_ may lead to better myopia control efficacy. This meaningful index could help in the evaluation and adjustment of lens parameters in a timely manner and minimize clinical trial and error time.

## Data Availability

The datasets used and/or analyzed during the current study are available from the corresponding author upon reasonable request.

## Abbreviations

*C*_*weighted defocus*_: Weighted Zernike defocus coefficient of the treatment zone
Ortho-K: Orthokeratology
AL: Axial length
HVID: Horizontal visible iris diameter
ACD: Anterior chamber depth
VST: Vision shaping treatment
CRT: Corneal refractive therapy
